# The Confusion Assessment Method Could Be More Accurate than the Memorial Delirium Assessment Scale for Diagnosing Delirium in Older Cancer Patients: An Exploratory Study

**DOI:** 10.3390/curroncol30090598

**Published:** 2023-09-06

**Authors:** Paula Llisterri-Sánchez, María Benlloch, Pilar Pérez-Ros

**Affiliations:** 1 Doctoral School, Catholic University of Valencia San Vicente Mártir, 46001 Valencia, Spain; 2 Coordinación Hospitalización, Hospital Intermutual de Levante, Km 11,7 CV-35, San Antonio de Benagéber, 46184 Valencia, Spain; 3 Department of Basic Biomedical Sciences, Catholic University of Valencia San Vicente Mártir, 46001 Valencia, Spain; 4 Department of Nursing, Universitat de València, Menendez Pelayo s/n, 46010 Valencia, Spain; 5 Frailty and Cognitive Impairment Research Group (FROG), Universitat de València, Menendez Pelayo s/n, 46010 Valencia, Spain

**Keywords:** cancer, older, delirium, assessment, prevalence

## Abstract

Background: Older people with cancer carry a high risk of delirium, an underdiagnosed syndrome due to its diagnostic complexity and often subtle presentation. Tools based on the Diagnostic and Statistical Manual of Mental Disorders (DSM) are available to different health professionals. Our aim is to assess the prevalence of delirium in older people with cancer in an inpatient unit and the accuracy of the Confusion Assessment Method (CAM) and Memorial Delirium Assessment Scale (MDAS). Methods: This exploratory, cross-sectional study included people aged 65 years or older with a diagnosis of cancer and admitted to the medical oncology unit from June 2021 to December 2022. The diagnostic accuracy of CAM and MDAS was analyzed against the gold standard medical diagnosis based on DSM-5 criteria by two medical oncologists. The cutoff point for the MDAS was determined using a receiver-operating characteristics (ROC) curve. Results. Among the 75 included patients (mean age 71.6 years, standard deviation 4.1; 52% males), the prevalence of delirium was 62.7%. The most prevalent types of cancer in patients with delirium were hematological and lung cancer. The scale with the highest diagnostic accuracy was the CAM, with a sensitivity of 100% and specificity of 86%, followed by the MDAS, with a sensitivity of 88% and specificity of 30%. The presence of cognitive impairment hindered the detection of delirium. Conclusions. The CAM scale was more accurate than the MDAS pre-existing cognitive impairment in our sample. Further studies are needed to analyze the diagnostic accuracy of delirium tools in older populations with cancer and in the presence of cognitive impairment.

## 1. Introduction

Delirium is a syndrome characterized by an acute and transitory alteration of a person’s mental state. Although its clinical presentation varies, it has an acute onset and fluctuating symptomatology, and it generally produces changes in attention, memory, orientation, and increased vulnerability to environmental stimuli. Delirium is a serious public health problem, associated with high morbidity and mortality, prolonged hospital stay, elevated health care costs, and reduced quality of life for patients and their caregivers. It is therefore important that healthcare professionals are trained to identify and manage delirium appropriately [1,2].

The etiology is multifactorial, with predisposing risk factors that include dementia, anxiety, depression, comorbidity, and sensory and functional deficits, along with precipitating factors like infections, anticholinergic drugs, benzodiazepines, pain, surgery, intensive care admissions, and sleep disorders. The highest risk of delirium occurs in older people due to the increased confluence of different risk factors [2].

In older people with cancer, these risk factors are further intensified by those related to drug toxicity, the use of harsh treatments and diagnostic tests, the presence of possible brain metastases, and cognitive dysfunction related to chemotherapy [3,4].

The prevalence of delirium in older cancer patients ranges from 22% to 57%, but it varies depending on the stage of the disease [5], the presence of other risk factors, and the patient’s underlying condition, among other variables. There is a higher prevalence in hospital settings than at home [5]. In the hospital setting, it should be noted that emergency and intensive care areas tend to have a higher incidence due to the existence of a greater number of invasive techniques, diagnostic tests, and the large number of staff working 24 h a day [2]. These actions are considered precipitating risk factors. In the rest of the hospitalization areas, there are also differences in surgical and medical areas, as well as in the different stages of the disease or type of cancer. The variability in the detection rates also depends on the difficulty of recognition according to the subtype of delirium. There are three subtypes of delirium, hyperactive, hypoactive, and mixed. The first is characterized by a state of hyper-alertness, with symptoms of hyperactivity, hallucinations and agitation. In contrast, hypoactive is characterized by a state of hypo alertness, with symptoms of lethargy and drowsiness. Mixed delirium is characterized by the presence of hyperactive and hypoactive symptoms. Hyperactive delirium is the most frequently detected due to its symptomatology, while hypoactive delirium is the most undetected due to the difficulty of detection, as these patients present fewer behavioral problems and are often perceived as cooperative [6].

Despite the high incidence and prevalence rates, the syndrome is underdiagnosed due to its diagnostic complexity and health professionals’ lack of proficiency in available detection methods [7].

The development of this syndrome leads to an increased mortality rate and a reduced survival prognosis. Since 50% of cases are reversible, correct diagnosis and appropriate treatment are essential. The diagnostic criteria are based on the Diagnostic and Statistical Manual of Mental Disorders, 5th edition (DSM-5), and the World Health Organization’s (WHO) International Classification of Diseases, 10th revision (ICD-10) [8].

Diagnosis is exclusively medical, so tools have been developed so that nursing staff, psychologists, and family members can quickly detect it [9]. The Confusion Assessment Method (CAM) is the world’s most widely used tool for diagnosing delirium and is easy to administer. It is based on two fundamental DSM-4 criteria and has high sensitivity and specificity [10]. Other instruments have been validated specifically for people with cancer to enable early detection. These include the Memorial Delirium Assessment Scale (MDAS) [11] or the Delirium Observation Screening (DOS) scale [12]. No tools have been validated exclusively in older people, although existing tools show adequate accuracy figures according to the literature [13]. Our aim is to estimate the prevalence of delirium in older inpatients with cancer and to assess the accuracy metrics of the CAM and MDAS scales against the gold standard medical diagnosis based on DSM-5 criteria.

## 2. Materials and Methods

### 2.1. Study Design, Setting, and Participants

This exploratory, cross-sectional study included people aged 65 years or older, diagnosed with cancer and admitted to the medical oncology unit of the Valencia General University Hospital from June 2021 to December 2022. The participants or their legal representatives agreed to participate in the study and signed informed consent. The project was approved by the hospital’s clinical research ethics committee (ref: 91/2021). Surgical patients admitted to the medical oncology unit, and those who refused to sign the informed consent form were excluded.

### 2.2. Sample Size Calculation

The sample size was determined based on the equation for estimating a proportion of the sample:

$$n\;\geq {\left(Z_{\left(1-\frac{\alpha }{2}\right)}\right)}^{2}\cdot \frac{p\cdot \left(1-p\right)}{d^{2}}$$

Assuming a prevalence of delirium in the older population of 22.6%, a sample size of 269 participants was calculated for an error of 5% and a confidence level of 95% [5].

During the 19-month recruitment period, a consecutive sample of 75 participants was obtained. Due to slow recruitment, an exploratory study was carried out to obtain preliminary results.

### 2.3. Procedure

Following hospital admission of an eligible patient, a nursing professional performed a complete assessment and review of the medical history. Variables collected included sociodemographic data (age, sex), along with the main oncological diagnosis and risk factors for delirium: dementia, incontinence, and type; use of bladder catheterization; urinary tract infection in the last six months; renal failure; falls in the previous month; dysphagia; hospitalizations in the last six months; visit to the emergency department (ED) in the last six months; number of drugs prescribed daily; and the use of anticholinergics, anxiolytics, antidepressants, and neuroleptics. In addition, the Mini Mental State Examination (MMSE) score was obtained on admission. The items on this scale are grouped into five domains, assessing orientation, fixation of knowledge in memory, concentration and calculation, deferred memory, and language and construction. The cutoff point for dementia is usually set at under 24 points [14]. During hospital admission, the nursing staff carried out daily assessments with the CAM and MDAS scales during each shift (morning, afternoon, and evening). The medical diagnosis was made using the DSM-5 diagnostic criteria. Two oncologists were responsible for the assessment of the DSM-5 criteria. The oncologists did not receive prior training.

The CAM scale has high sensitivity (94% to 100%) and specificity (90% to 95%) against the gold-standard medical diagnosis by a geriatrician. The short-form CAM evaluates four cognitive elements: (1) acute onset and fluctuating course; (2) inattention; (3) disorganized thinking; and (4) altered level of consciousness. To be diagnosed with delirium, a patient must demonstrate elements 1 and 2, as well as either 3 or 4 [10].

The MDAS is specifically designed to quantify the severity of delirium symptoms. It consists of 10 observer-rated items, each scored from 0 to 3 points and developed in line with DSM-4 diagnostic criteria for delirium. Items include level of consciousness, disorientation, short-term memory, digit span, attention, disorganized thinking, perceptual disturbances, delusions, psychomotor activity, and sleep–wake cycle disturbances. The scale integrates objective cognitive testing and assessment of behavioral symptoms. Repeated daily assessments are possible to capture short-term fluctuations in symptoms and to document treatment response. The MDAS yields an overall score ranging from 0 to 30, with a suggested cutoff of 13 points for delirium [11].

The DSM-5 criteria for delirium are as follow: (1) a disturbance in attention and awareness; (2) the disturbance develops over a short period of time, represents a change from baseline attention and awareness, and tends to fluctuate in severity during the course of a day; (3) an additional disturbance in cognition; (4) the disturbances in criteria 1 and 2 are not better explained by another pre-existing condition; (5) there is evidence from the history, physical examination, or laboratory findings that the disturbance is a direct physiological consequence of another medical condition, substance intoxication, or withdrawal [8].

### 2.4. Statistical Analysis

All the data entered into the database were independently verified by a second person. Descriptive statistics, in the form of the mean and standard deviation for normally distributed continuous variables and relative frequencies for categorical (qualitative) variables, were generated for all variables.

Diagnostic accuracy was assessed according to the scale validation cutoff point. In addition, the receiver operating characteristics (ROC) curve was used to determine the cutoff value and sensitivity, specificity, positive predictive value (PPV), and negative predictive value (NPV) of the MDAS scale.

Study data were entered in Microsoft Excel spreadsheets, followed by analysis using the SPSS statistical package (version 28.0, IBM Corp. Released 2015. IBM SPSS Statistics for Windows, Version 28.0. Armonk, NY, USA: IBM Corp.).

## 3. Results

Seventy-five patients (mean age 71.6 years, standard deviation 4.1; 52% males) were included. Over half (*n* = 47, 62.7%) experienced at least one episode of delirium, and 22 of these (46.8%) had more than one. The delirium group was on mean one year younger than the non-delirium group. There was a higher proportion of men with delirium, but no significant differences were found. Patients with delirium most commonly had hematological and lung cancer, and they showed a higher mean number of active drug treatments, a higher prevalence of treatment with anticholinergics and neuroleptics, and a higher number of ED visits (Table 1).

Cognition was analyzed with the MMSE scale, obtaining scores indicating severe cognitive impairment in both groups. The CAM results were positive for delirium in the 47 patients who were finally diagnosed by the oncologists with this syndrome as well as in 4 (14.3%) who were not. As for the MDAS scale, scores of 13 or more, indicating delirium, were obtained in 85.1% of those diagnosed, but also in 75% of those without delirium (Table 2).

The items of the MDAS scale were also analyzed individually, obtaining scores of 2 (indicating moderate impairment) in over half the sample for awareness, disorientation, attention, disorganized thinking, and sleep–wake cycle (Table 3).

Finally, the diagnostic accuracy of the MDAS and CAM scales was calculated with respect to the gold-standard DSM-5 criteria. The sensitivity of the CAM scale was higher than the MDAS (Table 4).

**Figure 1:**
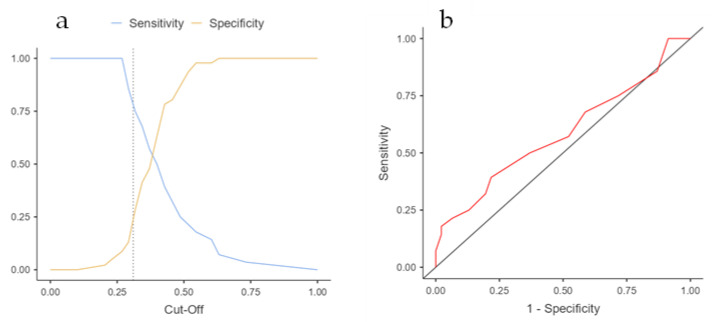
(**a**) Sensitivity and specificity of MDAS delirium diagnosis. (**b**) MDAS area under the curve.

The area under the curve for the MDAS was 0.59 (95% confidence interval [CI] 0.45 to 0.72) (Figure 1 and Table 3). The intersection point with the highest sensitivity and specificity was at a score of 15 points or more, with a sensitivity of 50% and a specificity of 63%.

## 4. Discussion

Older people with cancer are at higher risk for delirium due to the confluence of age- and cancer-related risk factors [15]. The prevalence of delirium in the sample analyzed was 62.7%, and the CAM scale presented greater diagnostic precision than the MDAS.

The prevalence of delirium varies in the oncology population due to the stage of the disease, the reason for hospitalization, and the hospital ward of admission, with higher rates in critical and palliative care units and lower ones in surgical admissions [5]. The method for detecting delirium can also influence its diagnosis since certain instruments require the assessor to have more training and experience; moreover, the time of day when the assessment takes place can also influence the result. The presence of cognitive impairment or dysfunction increases the risk of delirium, as do other factors [5]

Our results had a higher prevalence than other studies on the prevalence of delirium in medical oncology units and palliative care units, with rates from 22% to 57% [5]. This high prevalence is due to the inclusion of older people only, as well as the large presence of cognitive impairment in the study sample. Although a previous diagnosis of dementia did not exist in most participants, the MMSE assessment yielded very low scores, suggesting severe cognition impairment. The incidence and prevalence of cognitive impairment in older people with cancer is unclear, but different authors have called for cognitive screening in inpatients who eventually developed delirium, as well as those who did not [16]. Cognitive impairment and its more severe stage, dementia, frequently coexists with cancer in older people [17,18]. This situation can then be aggravated further with frequent admissions, invasive diagnostic procedures, and aggressive treatments with side effects, all of which exacerbate the risk of cognitive impairment, loss of functionality, and increased frailty [19,20]. Our results therefore suggest the need for a cognitive assessment, as this may impact treatment goals at each hospital admission in older people with these characteristics, as the risk of cognitive impairment is very high.

There is also evidence that people with cancer are at greater risk of suffering from cognitive impairment after starting chemotherapy or hormonal treatments, or due to the inflammatory process itself [21,22]. Cancer-related cognitive impairment has major consequences in the older population, such as loss of function and reduced decision-making ability, adherence to treatment, quality of life, and survival [23].

The delirium group was slightly younger than the non-delirium group. The older the age, the greater the risk of suffering delirium according to the literature. This risk factor is considered a low-intensity risk factor in the same way as renal failure, dehydration, and drowsiness. In our study, the presence of cognitive impairment could have interfered with the analysis, so studies with a larger sample and groups without cognitive impairment are needed. There was also a higher proportion of men in the delirium group than in the non-delirium group. Male gender is more related to delirium than female gender, and it is considered a medium-intensity risk factor. It is important to continue research in this population with delirium in order to implement prevention strategies [24].

The prevalence of different geriatric syndromes in older oncology patients underscores the need to perform a comprehensive geriatric assessment to prevent or mitigate the consequences derived from their onset [15,20,25]. The older population should be informed by a comprehensive understanding of everyone’s goals of care.

Although the need to screen for cognitive impairment in the older population as a whole is unclear, in those with cancer, there is no such controversy, as these patients are more vulnerable, and goals of care and decision making during the treatment process should be informed by a comprehensive understanding of each individual’s state [26]. Many simple tools exist and should be used to assess cognitive impairment, without neglecting the crucial information provided by the person themselves and their closest relatives [18].

Knowing the cognitive status of older patients could help health professionals to decide on the most appropriate procedure for detecting delirium, since screening is known to be useful for managing delirium in oncological surgical units [26]. One choice that clinicians face is the most appropriate screening tool for the population being served. There is currently no assessment tool validated exclusively in the older oncology population [13]. The CAM scale is the most widely used instrument worldwide for detecting delirium, including in cancer patients, and it has high sensitivity and specificity; however, it has not been validated in older people or in people with cancer [27]. The MDAS [11] and DOS [12] are the most common tools for assessing delirium in patients with advanced cancer [27]. The MDAS scale was developed to measure the severity of delirium symptoms as a way to diagnose delirium [11], with an optimal cutoff for diagnosis of ≥13 points. Several studies have analyzed the use of different cutoffs for detection, specifically, ≥7 points [28,29,30,31] and ≥9 points [32,33]. With the cutoff of ≥13 points, sensitivity in our sample was 87% and specificity was 25%. This may be due to the type of patient included. After analyzing the items of the MDAS scale in detail, high-to-moderate impairment was observed for disorganized thinking, disorientation, and alteration of consciousness. Raising the MDAS cut point for delirium to 15 still only gives a sensitivity of 0.50 and a specificity of 0.63.

Delirium and dementia have a special relationship in older adults; although they are two distinct entities, they are interconnected. Dementia is an insidious, chronic, and progressive loss of previously acquired cognitive ability. Delirium is an acute confusional state characterized by inattention, cognitive dysfunction, and an altered level of consciousness. People with dementia or cognitive impairment are at higher risk of developing delirium than the general population, and the occurrence of delirium is an independent risk factor for the subsequent development of dementia [2]. Furthermore, delirium in individuals with dementia may accelerate the trajectory of underlying cognitive impairment. In older people with cancer, this risk is further exacerbated [13]. There are few studies in humans that analyze the different tools for detecting delirium in people with dementia. The most reliable tools are the CAM and its intensive care version (CAM-ICU) [34], although analysis of each instrument indicates that all have their advantages and disadvantages [35]. Future research is needed to advance in the correct identification of delirium in moderate or severe dementia, especially in older people with cancer. Knowing the risk or existence of dementia or delirium in older cancer patients has implications during discussions about goals of care and treatment decisions. Older cancer patients with cognitive impairment or dementia have a greater number of predisposing factors for the onset of delirium. Moreover, invasive techniques such as blood extractions, radio diagnostic procedures, chemotherapy treatments, and the hospital environment itself (different schedules, noise, and light) can all act as precipitating factors [2,5]

In daily clinical practice, knowing the people at greatest risk of developing delirium during admission could prompt opportunistic screening and the implementation of prevention measures. Multicomponent interventions that promote an environment that minimizes risk factors in populations at increased risk of delirium in the hospital setting have health and cost benefits. Early detection and treatment minimize severity, length of hospital stay, chronicity, and mortality [36,37].

Limitations include the utilization of two oncologists rather than a psychiatrist to administer the gold standard DMS-5 for delirium. However, the oncologists were skilled clinicians comfortable with the care of older adults. It was not possible to analyze the severity, duration, or outcomes of delirium in our subjects.

## 5. Conclusions

The prevalence of delirium in hospitalized older people with cancer is 62.7%, and the CAM scale shows higher diagnostic accuracy than the MDAS in this exploratory study. The presence of cognitive impairment hinders the detection of delirium. Additional studies are needed to analyze the diagnostic accuracy of delirium tools in older people with cancer and underlying cognitive impairment.

## Figures and Tables

**Table 1:** Participant characteristics.

	Delirium (*n* = 47) *n* (%) *	No Delirium (*n* = 28) *n* (%) *	Total (*n* = 75) *n* (%) *	*p* Value
Age in years, mean (SD)	71.2 (4.2)	72.2 (3.9)	71.6 (4.1)	0.022 ^§^
Sex				
Male	26 (55.3)	13 (46.4)	39 (52.0)	0.46 ^‡^
Female	21 (44.8)	15 (53.6)	36 (48.0)	
Tumor site				
Breast	4 (8.5)	1 (3.6)	5 (6.7)	0.75 ^‡^
Skin	5 (10.6)	4 (14.3)	9 (12.0)	
Digestive cancer	14 (29.8)	6 (21.4)	20 (26.7)	
Hematological cancer	11 (23.4)	8 (28.6)	19 (25.3)	
Lung	11 (23.4)	6 (21.4)	17 (22.7)	
Gynecological cancer	0 (0)	1 (3.6)	1 (1.3)	
Prostate	2 (4.3)	2 (7.1)	4 (5.3)	
Risk factors				
Dementia	45 (95.7)	23 (82.1)	68 (90.7)	0.68 ^‡^
Incontinence	43 (91.5)	22 (78.6)	65 (86.7)	0.11 ^‡^
Urinary	18 (38.3)	6 (21.4)	24 (32.0)	0.15 ^‡^
Mixed	25 (53.2)	16 (57.1)	41 (54.7)	
Use of bladder catheter	17 (36.2)	6 (21.4)	23 (30.7)	0.18 ^‡^
Urinary infection in previous 6 months	7 (14.9)	7 (25.0)	14 (18.7)	0.28 ^‡^
Kidney failure	4 (8.5)	4 (14.3)	8 (10.7)	0.43 ^‡^
Falls in previous month	3 (6.4)	7 (25.0)	10 (13.3)	0.022 ^‡^
Dysphagia	24 (51.1)	5 (17.9)	29 (38.7)	0.040 ^‡^
Hospitalization in previous 6 months	43 (91.5)	24 (85.7)	67 (89.3)	0.58 ^‡^
ED visit in previous 6 months	43 (91.5)	25 (89.3)	68 (90.7)	0.75 ^‡^
Drug treatments				
Number of daily drugs, mean (SD)	11.8 (2.3)	10.9 (3.0)	11.5 (2.6)	0.14 ^§^
Anticholinergics	45 (95.7)	24 (85.7)	69 (92.0)	0.12 ^‡^
Anxiolytics	14 (29.8)	10 (35.7)	24 (32.0)	0.56 ^‡^
Antidepressants	6 (12.8)	1 (3.6)	7 (9.3)	0.19 ^‡^
Neuroleptics	18 (38.3)	12 (42.9)	30 (40.0)	0.70 ^‡^

* Unless otherwise noted; ^‡^ chi-square test; ^§^ student’s *t*-test.

**Table 2:** Cognition and delirium assessment tools scores.

Variables	Delirium (*n* = 47)	No Delirium (*n* = 28)	Total (*n* = 75)	*p* Value
MMSE, points, mean (SD)	7.6 (2.4)	8.4 (2.2)	7.9 (2.3)	0.23 *
CAM positive, *n* (%)	47 (100)	4 (14.3)	51 (68.0)	<0.001 ^†^
MDAS, points, mean (SD)	16.63 (3.71)	14.8 (4.5)	15.9 (4.1)	0.22 *
MDAS ≥ 13 points, *n* (%)	40 (85.1)	21 (75.0)	61 (81.3)	0.19 ^†^

CAM: Confusion Assessment Method; MDAS: Memorial Delirium Assessment Scale. MMSE: Mini Mental State Examination. * Student’s *t*-test; ^†^ chi-square test.

**Table 3:** MDAS scores in the total sample (*n* = 75), *n* (%).

	Severity of Symptoms				
MDAS Items	None = 0	Mild = 1	Moderate = 2	Severe = 3	Mean (SD)
Awareness	10 (13.5)	7 (9.2)	43 (58.1)	14 (18.9)	1.82 (0.89)
Disorientation	3 (4.1)	19 (25.7)	41 (55.4)	11 (14.9)	1.84 (0.73)
Memory	19 (25.7)	8 (10.8)	33 (44.6)	14 (18.9)	1.57 (1.07)
Digit span	33 (44.6)	9 (12.2)	21 (28.9)	11 (14.9)	1.14 (1.15)
Attention	5 (8.1)	14 (18.9)	38 (51.4)	16 (21.6)	1.86 (0.85)
Disorganized thinking	7 (9.5)	13 (17.6)	39 (52.7)	15 (20.3)	1.84 (0.86)
Perception	24 (32.4)	7 (9.5)	32 (43.2)	11 (14.9)	1.41 (1.09)
Delusions	31 (41.9)	6 (8.1)	27 (36.5)	10 (13.5)	1.22 (1.14)
Psychomotor activity	26 (35.1)	8 (10.8)	28 (37.8)	12 (16.2)	1.35 (1.13)
Sleep–wake cycle	8 (10.8)	1 (1.4)	54 (73)	11 (14.9)	1.92 (0.77)

MDAS: Memorial Delirium Assessment Scale.

**Table 4:** Accuracy of MDAS and CAM in the sample.

	Sensitivity % (95% CI)	Specificity % (95% CI)	PPV % (95% CI)	NPV % (95% CI)
MDAS ≥ 13 (gold DSM)	87 (77.2–96.7)	25 (9.2–41.3)	66 (52.3–79.7)	54 (35.5–72.5)
CAM (gold DSM)	100 (100–100)	86 (73.1–98.8)	92 (81.9–99)	100 (100–100)

CAM: Confusion Assessment Methods; CI: Confidence interval; DSM: Diagnostic and Statistical Manual of Mental Disorders; MDAS: Memorial Delirium Assessment Scale; NPV: negative predictive value; PPV: positive predictive value.

## Data Availability

Data available on request from the authors.
